# Misfit and fracture load of implant-supported monolithic crowns in zirconia-reinforced lithium silicate

**DOI:** 10.1590/1678-7757-2016-0233

**Published:** 2017

**Authors:** Rafael Soares GOMES, Caroline Mathias Carvalho de SOUZA, Edmara Tatiely Pedroso BERGAMO, Dimorvan BORDIN, Altair Antoninha DEL BEL CURY

**Affiliations:** 1 Universidade Estadual de Campinas, Faculdade de Odontologia de Piracicaba, Departamento de Prótese e Periodontia, Piracicaba, SP, Brasil.; 2 Universidade Estadual de Campinas, Faculdade de Odontologia de Piracicaba, Departamento de Odontologia Restauradora, Piracicaba, SP, Brasil.

**Keywords:** Dental materials, Dental prosthesis, X-Ray microtomography

## Abstract

Zirconia-reinforced lithium silicate (ZLS) is a ceramic that promises to have better mechanical properties than other materials with the same indications as well as improved adaptation and fracture strength.

**Objective:**

In this study, marginal and internal misfit and fracture load with and without thermal-mechanical aging (TMA) of monolithic ZLS and lithium disilicate (LDS) crowns were evaluated.

**Material and methods:**

Crowns were milled using a computer-aided design/computer-aided manufacturing system. Marginal gaps (MGs), absolute marginal discrepancy (AMD), axial gaps, and occlusal gaps were measured by X-ray microtomography (n=8). For fracture load testing, crowns were cemented in a universal abutment, and divided into four groups: ZLS without TMA, ZLS with TMA, LDS without TMA, and LDS with TMA (n=10). TMA groups were subjected to 10,000 thermal cycles (5-55°C) and 1,000,000 mechanical cycles (200 N, 3.8 Hz). All groups were subjected to compressive strength testing in a universal testing machine at a crosshead speed of 1 mm/min until failure. Student’s t-test was used to examine misfit, two-way analysis of variance was used to analyze fracture load, and Pearson’s correlation coefficients for misfit and fracture load were calculated (α=0.05). The materials were analyzed according to Weibull distribution, with 95% confidence intervals.

**Results:**

Average MG (p<0.001) and AMD (p=0.003) values were greater in ZLS than in LDS crowns. TMA did not affect the fracture load of either material. However, fracture loads of ZLS crowns were lower than those of LDS crowns (p<0.001). Fracture load was moderately correlated with MG (r=-0.553) and AMD (r=-0.497). ZLS with TMA was least reliable, according to Weibull probability.

**Conclusion:**

Within the limitations of this study, ZLS crowns had lower fracture load values and greater marginal misfit than did LDS crowns, although these values were within acceptable limits.

## Introduction

The evolution of ceramic systems has been guided by efforts to enhance their strength and aesthetics^32^. The use of zirconia seemed to solve the problem of resistance in these systems, but the aesthetic quality of this material is less than desirable^2^. In the effort to obtain an aesthetic and strong material, a ceramic with 10% zirconia added to lithium silicate was recently developed and released^19^. Named zirconia-reinforced lithium silicate (ZLS), this material was designed for exclusive use with computer-aided design/computer-aided manufacturing (CAD/CAM) systems. Its manufacturer claims that it is an outstanding aesthetic material, with more strength and easy milling ability compared with lithium disilicate (LDS), generating optimized edge stability. Nevertheless, few studies supporting these features have been published^6,19,30^.

LDS monolithic crowns have been used with success^26^. Monolithic crowns withstand greater resistance than bi-layered crowns, and can be used in regions with greater masticatory forces^24^. With the application of extrinsic staining techniques, LDS has been established as an aesthetic and strong material^7^. The use of ZLS in combination with CAD/CAM technology appears to be another option for restorative treatments with similar indications and requirements as for LDS^30^.

CAD/CAM technology has facilitated restorative prosthetic treatment for clinicians and patients, decreasing restoration placement and overall chair times^12^. Although the use of this technique has widely spread, misfit values can differ among restorations made with the same system and impression technique due to material composition^1,12,14,16^. Material hardness can interfere with fit results, as harder materials can make the milling process difficult, leaving crowns susceptible to small fractures in very thin regions, such as cervical areas^14,17,25,27^; thick and irregular cement lines can also compromise fit^11,28^. In addition, some studies have shown that the worse the adaptation of the crown, the lower its resistance^28,33^.

Threshold values for marginal and internal misfit remain a matter of debate. The most commonly accepted value is around 120 μm, but values of 50 μm and 200 μm have also been reported^3,9,15^. Some authors have claimed that thick cement layers may lead to increased cement dissolution, microleakage, localized stress accumulation, and reduction of fracture strength^20,21,28^.

The clinical success of a restoration can depend on many factors, including fracture load, which can be affected by marginal/internal fit and the ability to withstand cyclic loading^1,31,33^. These properties need to be investigated in ZLS; studies testing the advantages attributed to this material remain scarce. This study was conducted to evaluate marginal and internal misfit, fracture load with and without thermal-mechanical aging (TMA), and reliability of ZLS compared with LDS. The hypothesis was that the fit and fracture load of ZLS would be superior to those of LDS.

## Material and methods

### Specimens fabrication

Using a three-dimensional (3D) optical scanning device (Ceramill Map400, Amann Girrbach, Koblach, Vorarlberg, Austria), a 3D digital model of a morse taper universal abutment (Munhão Universal, Intraoss, Itaquaquecetuba, São Paulo, Brazil) with a 4.5 mm diameter, 6 mm height, and 2.5 mm collar height was obtained. From this model, and regarding the anatomy of a mandibular first molar, a monolithic crown was drawn using CAD software (Ceramill Mind, Amann Girrbach). From this CAD model, 20 ZLS crowns (Suprinity, Vita Zahnfabrik, Bad Säckingen, Baden-Württemberg, Germany) and 20 LDS crowns (IPS e.max CAD, Ivoclar Vivadent, Schaan, Liechtenstein) were milled (Ceramill Motion 2, Amann Girrbach). Integrity of crown margins was examined by scanning electron microscopy (SEM) (JSM-5600LV, Jeol, Boston, Massachusetts, USA).

### Marginal/internal misfit

**Figure 1:**
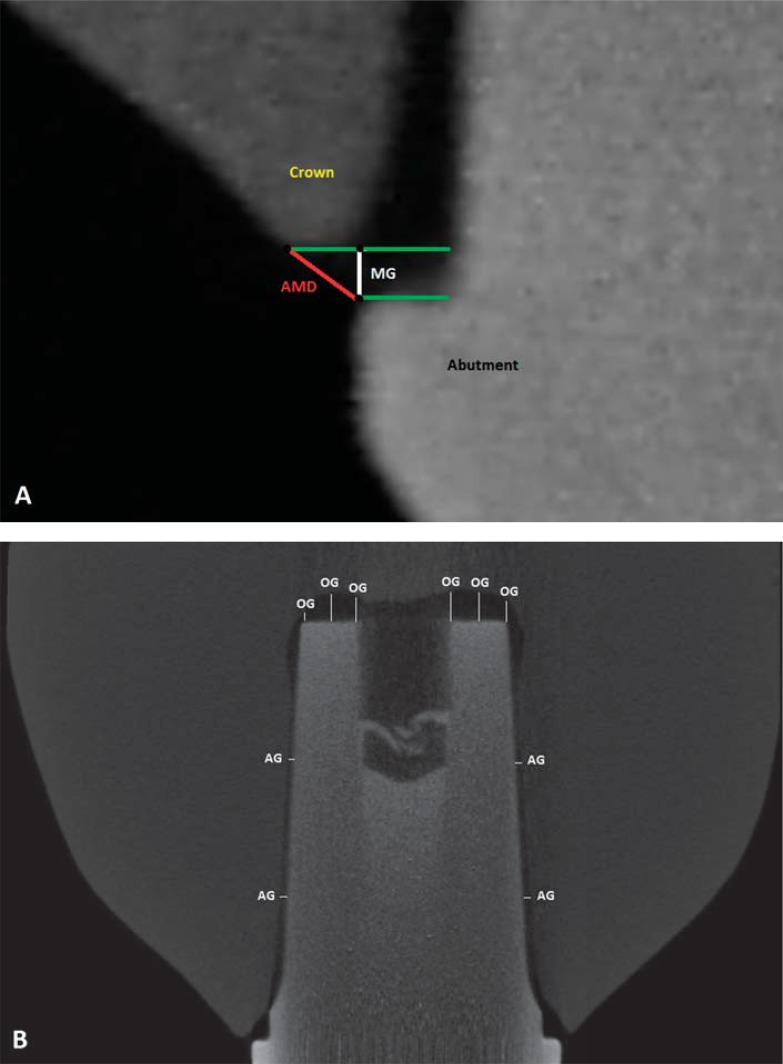
A: Points used for the measurement of marginal gap (MG) and absolute marginal discrepancy (AMD). B: Points used for the measurement of axial gap (AG) and occlusal gap (OG)

Marginal and internal misfit of eight crowns of each material was evaluated using X-ray microtomography (μCT) (Skyscan 1176, Bruker, Billerica, Massachusetts, USA). Each crown–universal abutment set was twice wrapped for an adhesive tape that ran through on the occlusal surface to the abutment’s base, avoiding any displacement, and positioned perpendicular to the X-ray source. The parameters used for image acquisition were 80 kV, 1400 ms exposure time, 0.5° rotation step, 360° rotation, and 1 mm Cu + Al filter. Seven hundred twenty images were obtained in each scan and reconstructed using NRecon software (Bruker). Using DataView software (Bruker), central coronal and sagittal slices were isolated from the reconstructed images. Using CTAn software (Bruker), the averages of the values obtained on each axis were calculated. Following Holmes, et al.^8^ (1989), four locations (centers of the buccal, lingual, mesial, and distal faces) were selected for the evaluation of marginal gaps (MGs) and absolute marginal discrepancy (AMD; Figure 1A). Ten locations on each of the two slice types [four on the axial walls for the evaluation of axial gaps (AGs) and six on the occlusal walls for the evaluation of occlusal gaps (OGs) (Figure 1B)] were used for the evaluation of internal misfit^3,12,13^.

### Fracture load

Prior to cementation, the crowns (n=20 of each material) were rinsed in 98% alcohol for 1 minute in an ultrasonic bath. Their external surfaces were then protected with wax (New Wax, Technew, Rio de Janeiro, Rio de Janeiro, Brazil), and the intaglio surfaces were conditioned with 5% hydrofluoric acid (Condac porcelana, FGM, Joinville, SC, Brazil) for 20 seconds. The excess gel was removed with a water jet, and the crowns were washed again in the ultrasonic bath with 98% alcohol for 3 minutes. A thin layer of silane coupling agent (Prosil, FGM) was then applied to the intaglio surfaces and allowed to act for 60 seconds; excess silane was volatilized with an air jet. The crowns were cemented into abutments that had been tightened into implant analogs^28^ (Titaoss Max CM Analog, Intraoss) with 32 N.cm torque (TQ8800, Lutron, Taipei, Taiwan) using a dual-cure resin composite cement (Panavia F, Kuraray Noritake Dental Inc., Okayama, Tokyo, Japan), and photopolymerized for 20 seconds/face using an LED source with 1000 mW/cm^2^ light intensity (VALO, Ultradent Products Inc, South Jordan, Utah, USA).

The analogs were embedded in polyurethane resin (F160, Axson Technologies, Saint Ouen I’Aumône, France) at a 30° angle (based on ISO 14801:2007)^10^ in a metal matrix (20 mm diameter, 20 mm height). Twenty crowns were used as the experimental groups and the other 20 crowns served as controls. Ten crowns of each material were subjected to 1 million mechanical cycles (200 N load, 3.8 Hz frequency; ER-1300, ERIOS, São Paulo, SP, Brazil), with 10,000 thermal cycles (MSCT-3e, Elquip, São Carlos, SP, Brazil) in alternating water baths with temperatures of 5°C and 55°C (30 seconds each with a 5 second interval), amounting to about 65 seconds per cycle.

For fracture load testing, a mechanical load was applied (Instron 4411, Instron, Norwood, Massachusetts, USA) with a stainless-steel hemispherical indenter (5 mm diameter) to the occlusal surface of each crown at a crosshead speed of 1 mm/min until failure. Failure was defined as chipping or catastrophic fracture of the crown. After fracture, all samples were submitted to fractographic analysis by SEM to identify the origin of failure^4,22,23^.

### Statistical analysis

Misfit and fracture load data were analyzed using the IBM SPSS Statistics 20 software (IBM Corp., Armonk, New York, USA) with a significance level of 5% (α=0.05). Normal data distribution was confirmed by the Shapiro–Wilk test for misfit and fracture load variables. All misfit outcomes (MG, AMD, AG, and OG) were separately compared between materials using Student’s t-tests. The means of fracture loads in the two groups were examined using two-way ANOVA. The power obtained with the current sample size in both analyses exceeded 90%. Pearson’s correlation coefficients were calculated to determine whether a linear relationship existed between misfit and fracture load. The Weibull distribution was examined using SAS software (SAS Institute Inc., Cary, North Carolina, USA), with a 95% confidence interval, to characterize the reliability of sample survival based on the applied load.

## Results

**Figure 2:**
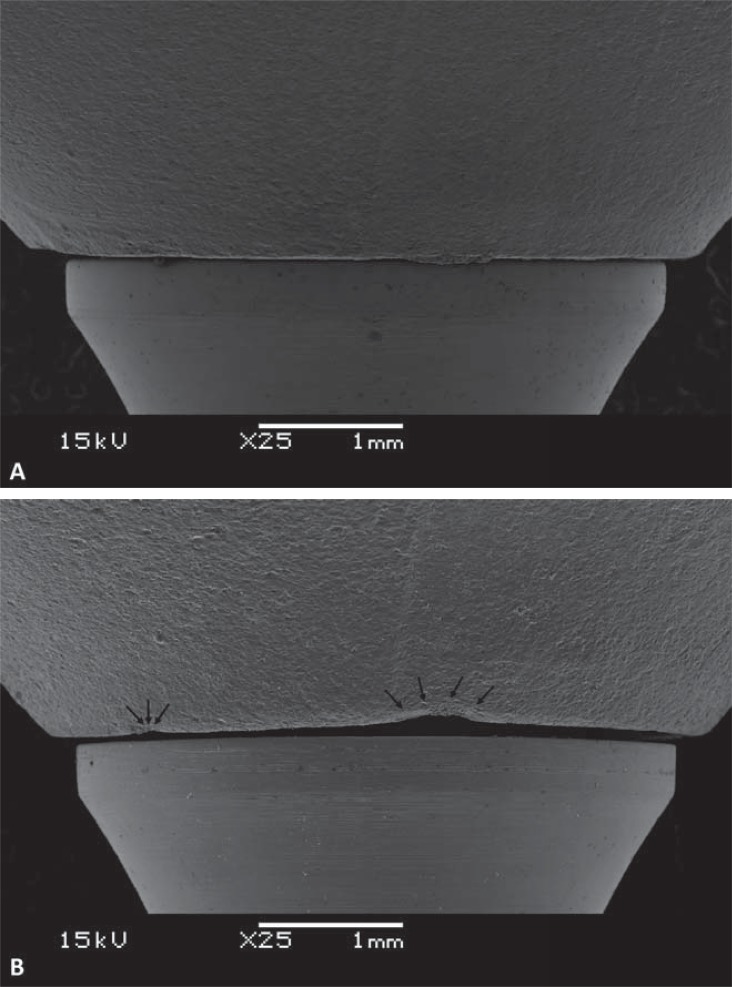
A: Cervical area of the lithium disilicate crown with no chipping. B: Cervical area of the zirconia-reinforced lithium silicate crown, showing chipping at the edge (arrows)

MG (p<0.001) and AMD (p=0.003) values were lower for LDS crowns than for ZLS crowns. No significant difference in AG or OG values was observed (Table 1). Chipping in the cervical region occurred during the milling process in some crowns (Figure 2A and B).

**Table 1:** Marginal gap (MG), absolute marginal discrepancy (AMD), axial gap (AG), and occlusal gap (OG) values (µm; n=8; mean±standard deviation)

Group	MG	AMD	AG	OG
LDS	41.45±18.11^a^	180.67±23.23^a^	96.07±30.19^a^	255.80±65.05^a^
ZLS	101.86±32.12^b^	235.54±35.75^b^	100.09±23.83^a^	252.68±35.18^a^

LDS, lithium disilicate; ZLS, zirconia-reinforced lithium silicate. Different superscripted letters indicate significant differences between materials (Student’s t-test, p<0.05).

**Figure 3:**
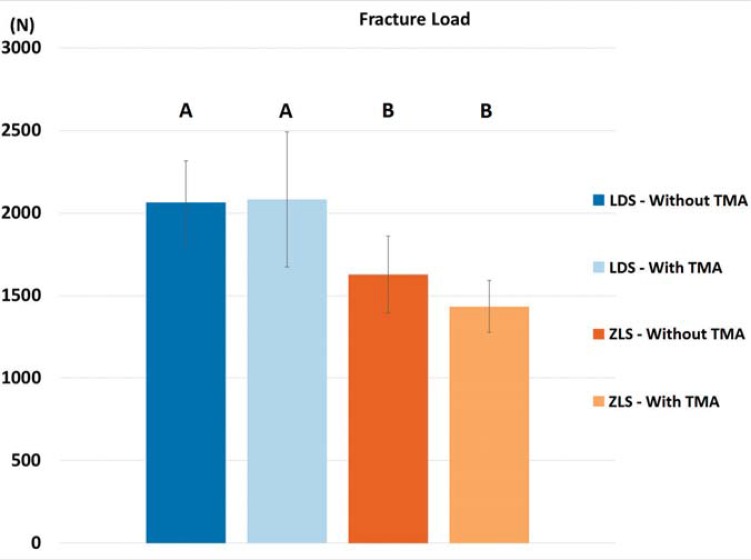
Mean fracture loads of LDS and ZLS crowns before and after thermal-mechanical aging. Bars indicate standard deviations and different letters indicate significant differences (two-way ANOVA, p<0.05)

Material type significantly influenced the fracture loads of monolithic crowns (p<0.001), whereas the TMA procedure did not significantly change the fracture load (Table 2). Mean fracture load values without and with TMA were 2062.73±251.74 N, and 2083.23±407.97 N, respectively, for LDS crowns and 1626.83±233.98 N and 1433.54±156.46 N, respectively, for ZLS crowns (Figure 3). Fracture load was not significantly correlated with OG (r=0.185, p>0.05) or AG (r=-0.237, p>0.05), but it showed moderate negative correlations with MG (r=-0.553, p=0.026) and AMD (r=-0.49, p=0.05).

**Table 2:** Two-way ANOVA (2x2) of material, aging condition, and interactions between these variables

Source	SS	df	MS	F	P
Material	2946302.1	1	2946302.1	38.13	.000
Aging condition	74643.6	1	74643.6	0.97	.332
Material*Aging condition	114260.1	1	114260.1	1.48	.232
Error	2781347.9	36	77259.7		

SS, Sum of Squares; df, degree freedom; MS, Mean Square

**Figure 4:**
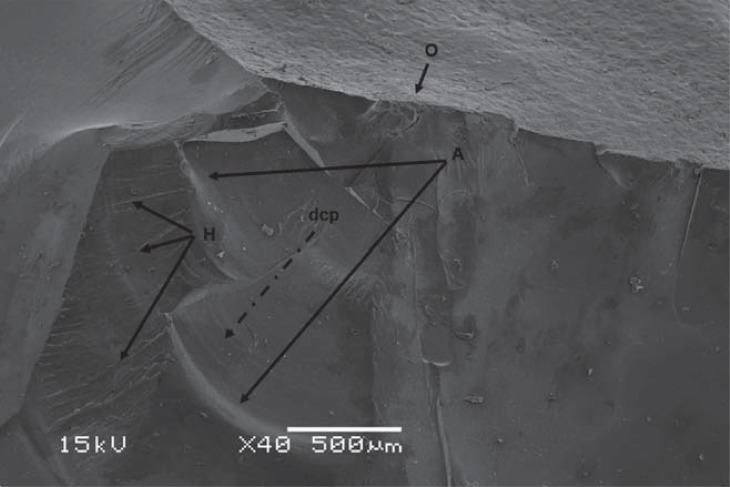
Fractographic image showing fracture origin (O), direction of crack propagation (dcp), arrest lines (A), and hackle lines (H)

**Figure 5:**
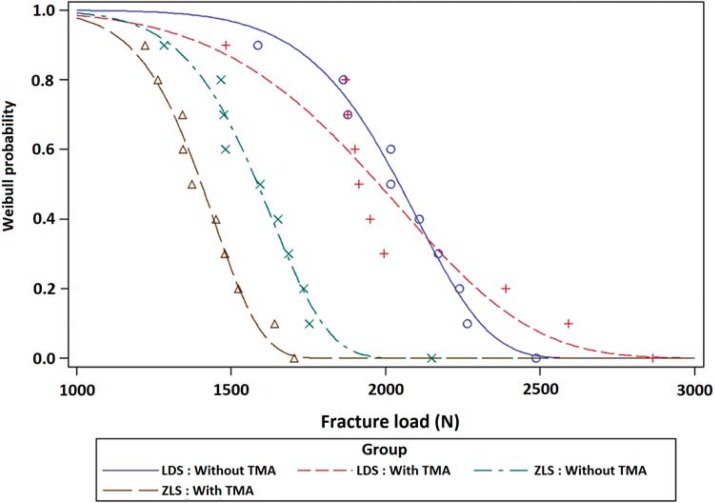
Weibull distribution showing failure probability according to the applied load

Crowns presented catastrophic failure, exposing the abutment. Hackle lines, commonly formed when cracks grow rapidly, and arrest lines, which indicate the direction of crack propagation, showed that crack propagation originated from the load point (Figure 4). The Weibull distribution showed overlapping of the two LDS aging conditions, and higher reliability than observed for ZLS crowns in both conditions (Figure 5).

## Discussion

Studies on internal and marginal misfit of monolithic ZLS crowns are scarce. The use of a non-destructive measurement technique, such as μCT, has the great advantage of enabling evaluation of not only the marginal area, but also the intaglio surface, while preserving samples for further analyses (e.g., fracture load) with no effect on the results. It may, however, be considered a costly and slow technique^13,16^.

In this study, ZLS crowns showed higher misfit in the cervical region than did LDS crowns, which presented misfit values compatible with those published previously^12-14^. One reason for misfit in the cervical region could be that a small amount of internal space obstructs the settling of the crown^1^, but this was not observed in this study, since the two materials produced similar internal adaptation values. A second hypothesis is that this difference could be due to the occurrence of very small fractures during milling of the cervical region in ZLS crowns^14^. The results of this study support this hypothesis, as SEM images showed more irregularity in the cervical regions of ZLS crowns compared with LDS crowns, which may have increased the average marginal misfit of ZLS crowns. In this study was considered that there are no differences among abutment sizes, which could interfere also in the measurements outcomes.

Margin inaccuracy may be related to the increased brittleness index and chipping factor of ZLS, resulting in greater marginal misfit, probably due to the presence of zirconia in the microstructure^19,27^. The chipping factor (%) is the ratio of the total amount of chipping around the marginal circumference of the restoration multiplied by 100^27^. It is positively related to the brittleness index, which is a ratio of hardness and fracture toughness. A higher hardness value and lower fracture toughness value increase the brittleness index of a material, indicating that it is more prone to chipping^27^. According to the manufacturers of the materials used in this study, ZLS has a higher hardness value than does LDS in crystallized mode (7000 vs. 5600 MPa), and a lower fracture toughness value (2.00 vs. 2.25 MPa m^-0^). However, no study has examined these properties in the pre-crystalized phase, in which these restorations are milled.

The occlusal and axial walls of ZLS and LDS crowns showed no difference in misfit. These thick, regular areas may be less vulnerable to microfracture, thus supporting the hypothesis that thin regions are more susceptible to damage during the milling process. Although ZLS presented a greater difference in marginal misfit compared with LDS, this degree of misfit is within the clinically acceptable limits of 120 µm according to most studies^1,3,11-16,21,28^. In addition, the greatest discrepancy did not seem to compromise the mechanical behavior of the material a lot, since moderate negative correlations were observed between fracture load and MG and AMD.

Internal misfit is less commonly evaluated than marginal misfit. Gaps >122 µm in axial walls may reduce the fracture strength of a crown^16,17,25,27^. In the present study, AG values for both materials were <122 µm, and AG was not correlated with fracture load. Another area of internal analysis is the occlusal wall, where misfit exceeding 450 µm may compromise cementation due to polymerization shrinkage^16^. In this study, OG values were <450 µm, within the clinically acceptable range for general internal misfit, and did not differ between materials^12^.

Fracture loads were lower for ZLS crowns than for LDS crowns, with and without TMA. The presence of zirconia in the microstructure seems to increase material hardness, making it more prone to chipping during milling^19^. Chipping can worsen the adaptation of the material and indirectly compromise fracture loads^20^. A negative linear correlation between misfit and fracture loads was detected in this study. TMA did not significantly affect fracture load, contrary to several previous reports that aging influences the resistance of dental ceramics, although the Weibull probability curves showed that ZLS is affected more than LDS^5,31,33^. However, comparison of the present results with those of other studies should be done with caution, given the poor standardization of loads, frequency, number of cycles, and substrates. In addition, Weibull probability can be unreliable in small samples (mainly those with n<10)^18^, although confidence intervals were high in the present analysis.

Both materials tested in this study resisted loads larger than maximum relative values of bite force found in the literature (~880 N)^29^. However, the fracture load test does not mimic failures occurring when crowns are in clinical use^4,5,33^. The fractographic analysis conducted in the present study showed crack propagation from the load point to the cervical area in all samples, opposite the direction of propagation in clinical situations^4^. Thus, we cannot confirm that the results of the present study represent the real clinical behavior of these materials.

The results of this study suggest that ZLS does not outperform LDS in terms of fracture load, fit quality, or reliability. However, the effectiveness of this material must be confirmed in clinical trials.

## Conclusion

Within the limitations of this study, ZLS crowns had lower fracture load values and greater marginal misfit than did LDS crowns. Despite these differences, all values were within the limits considered to be acceptable. Thus, both materials comply with the indication criteria.
